# Food Costs of a Low-Fat Vegan Diet vs a Mediterranean Diet: A Secondary Analysis of a Randomized Clinical Trial

**DOI:** 10.1001/jamanetworkopen.2024.45784

**Published:** 2024-11-18

**Authors:** Hana Kahleova, Macy Sutton, Cristina Maracine, Daniel Nichols, Pablo Monsivais, Richard Holubkov, Neal D. Barnard

**Affiliations:** 1 Physicians Committee for Responsible Medicine, Washington, DC; 2 School of Public Health, George Washington University, Washington, DC; 3 The Norton College of Medicine, SUNY Upstate Medical University, Syracuse, New York; 4 Department of Nutrition and Exercise Physiology, Elson S. Floyd College of Medicine, Washington State University, Spokane; 5 School of Medicine, University of Utah, Salt Lake City; 6 School of Medicine and Health Sciences, George Washington University, Washington, DC

## Abstract

This ad hoc secondary analysis of a randomized clinical trial compares the food costs in the United States of a low-fat vegan diet and a Mediterranean diet.

## Introduction

Vegan and Mediterranean diets have health benefits, but affordability may present a barrier to dietary change. This ad hoc secondary analysis of a randomized crossover trial comparing vegan and Mediterranean diets^1^ assessed the food costs of these 2 diets.

## Methods

This trial, conducted from February to October 2019, was conducted in accordance with the Declaration of Helsinki, follows the Consolidated Standards of Reporting Trials (CONSORT) reporting guideline, and was approved by the Advarra institutional review board.^1^ All participants provided written informed consent. Participants with overweight were randomized in a 1:1 ratio to a low-fat vegan or a Mediterranean diet for 16 weeks, separated by a 4-week washout. The vegan diet consisted of fruits, vegetables, grains, and legumes. The Mediterranean diet was based on the PREDIMED protocol^2^ (trial protocol in Supplement 1). No instructions on food costs were given. A 3-day dietary record (2 weekdays and 1 weekend day) was completed by participants at weeks 0, 16, 20, and 36 and analyzed by a registered dietitian certified in the Nutrition Data System for Research.^3^ For the food cost assessment, intakes from dietary records were linked to the US Department of Agriculture Thrifty Food Plan, 2021,^4^ a database of national food prices, which are calculated from data collected for the Consumer Price Index. Two independent reviewers (C.M. and D.N.), blinded to group assignment, linked the database prices with food groups from the dietary analysis software. Linking accuracy was verified by a senior researcher (P.M.), also blinded to group assignment.

Statistical analysis was performed using SAS version 9.4 in July 2024 on a per-protocol basis for all participants with complete data across all time points by a statistician blinded to dietary interventions. Treatment effect size was quantified by comparing changes from baseline (from week 0 to 16 and from week 20 to 36) in vegan vs Mediterranean diets, using paired *t* tests. All results are presented as mean values with 95% CIs. *P* values were 2-sided and deemed significant at *P* < .05

## Results

Of 506 people screened by telephone, 62 (mean [SD] age, 57.4 [9.8] years; 14 [23%] men and 48 [77%] women) met participation criteria and were randomly assigned to start the vegan (n = 30) or Mediterranean (n = 32) diet (eFigure in Supplement 2). Total food costs decreased on the vegan diet by 19% (−$1.8/d [95% CI, −$2.6/d to −$1.0/d]; *P* < .001), compared with no significant change on the Mediterranean diet ($0.6/d [95% CI, −$0.3/d to $1.6/d]; *P* = .20); the difference between food costs on both diets was 25% (effect size, −$2.4/d [95% CI, −$3.6/d to −$1.3/d]; *P* < .001) (Figure; Table). This decrease in costs on the vegan diet was mainly associated with savings on meat (−$2.9/d [95% CI, −$3.6/d to −$2.1/d]; *P* < .001), dairy (−$0.5/d [95% CI, −$0.8/d to −$0.2/d]; *P* < .001), and added fats (−$0.5/d [95% CI, −$0.6/d to −$0.3/d]; *P* < .001). These savings outweighed the increased spending on vegetables ($0.5/d [95% CI, $0.0/d-$1.1/d]; *P* = .03), grains (95% CI, $0.3/d [95% CI, $0.0/d-$0.6/d]; *P* = .04), and meat alternatives ($0.5/d [95% CI, $0.2/d-$0.7/d]; *P* = .001) on the vegan diet (Table).

**Figure.**
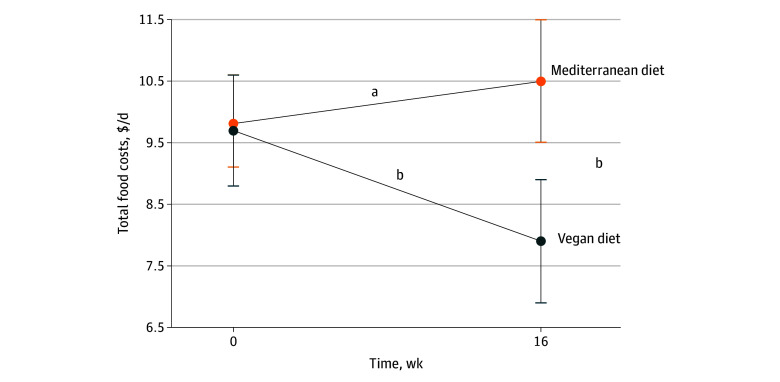
Changes in Total Food Costs on the Vegan and Mediterranean Diets The data are shown as mean values with 95% CIs. ^a^*P* = .20. ^b^*P* < .001.

**Table.** Changes in Food Costs From Specific Food Groups During the Study Comparing a Mediterranean and a Low-Fat Vegan Diet^a^

Variable	Mediterranean diet			Vegan diet			Treatment effect size	*P* value
	Baseline	Final	Change	Baseline	Final	Change		
Economic costs from specific food groups, $/d (95% CI)								
Total food costs	9.8 (9.1 to 10.6)	10.5 (9.5 to 11.5)	0.6 (−0.3 to 1.6)	9.7 (8.8 to 10.6)	7.9 (6.9 to 8.9)	−1.8 (−2.6 to −1.0)^b^	−2.4 (−3.6 to −1.3)	<.001
Fruit	1.1 (0.8 to 1.3)	1.2 (1.0 to 1.5)	0.2 (−0.1 to 0.4)	0.9 (0.7 to 1.1)	1.3 (1.0 to 1.6)	0.4 (0.2 to 0.7)^c^	0.2 (−0.2 to 0.6)	.24
Vegetables	2.4 (2.1 to 2.7)	2.9 (2.4 to 3.4)	0.5 (0.1 to 0.9)^d^	1.9 (1.7 to 2.2)	3.0 (2.5 to 3.5)	1.0 (0.7 to 1.4)^b^	0.5 (0.0 to 1.1)	.03
Grains	1.5 (1.3 to 1.7)	1.1 (1.0 to 1.3)	−0.3 (−0.6 to −0.1)^d^	1.4 (1.2 to 1.6)	1.4 (1.2 to 1.6)	−0.0 (−0.2 to 0.2)	0.3 (0.0 to 0.6)	.04
Whole grains	0.4 (0.3 to 0.5)	0.5 (0.4 to 0.6)	0.1 (0.0 to 0.2)^d^	0.4 (0.3 to 0.5)	0.5 (0.4 to 0.6)	0.2 (0.1 to 0.3)^c^	0.0 (−0.1 to 0.2)	.75
Some whole grains	0.1 (0.1 to 0.2)	0.1 (0.0 to 0.1)	−0.0 (−0.1 to 0.0)	0.1 (0.0 to 0.1)	0.1 (0.1 to 0.2)	0.0 (−0.0 to 0.1)	0.1 (−0.0 to 0.2)	.06
Refined grains	1.0 (0.8 to 1.2)	0.6 (0.4 to 0.7)	−0.4 (−0.7 to −0.1)^c^	1.0 (0.8 to 1.1)	0.7 (0.6 to 0.9)	−0.2 (−0.5 to −0.0)^d^	0.2 (−0.1 to 0.5)	.26
Meat	2.2 (1.8 to 2.7)	2.7 (2.2 to 3.1)	0.4 (−0.2 to 1.0)	2.6 (2.2 to 3.0)	0.1 (0.0 to 0.3)	−2.5 (−2.9 to −2.1)^b^	−2.9 (−3.6 to −2.1)	<.001
Red meat	0.4 (0.2 to 0.5)	0.1 (0.0 to 0.2)	−0.3 (−0.5 to −0.1)^b^	0.6 (0.3 to 0.8)	0.0 (0.0 to 0.1)	−0.5 (−0.8 to −0.3)^c^	−0.2 (−0.6 to 0.1)	.11
White meat	1.7 (1.3 to 2.1)	2.4 (1.9 to 2.9)	0.7 (0.2 to 1.3)^d^	1.6 (1.3 to 1.9)	0.0 (−0.0 to 0.1)	−1.6 (−1.9 to −1.2)^b^	−2.3 (−3.0 to −1.6)	<.001
Fried meat	0.0 (0.0 to 0.1)	0.0 (0.0 to 0.0)	−0.0 (−0.1 to 0.0)	0.1 (0.0 to 0.2)	0.0 (0.0 to 0.0)	−0.1 (−0.2 to −0.0)^d^	−0.1 (−0.2 to 0.0)	.08
Processed meat	0.2 (0.1 to 0.2)	0.1 (0.1 to 0.2)	−0.0 (−0.1 to 0.1)	0.3 (0.2 to 0.4)	0.0 (0.0 to 0.1)	−0.3 (−0.4 to −0.2)^b^	−0.2 (−0.4 to −0.1)	.001
Eggs	0.2 (0.1 to 0.2)	0.2 (0.1 to 0.2)	−0.0 (−0.1 to 0.1)	0.2 (0.1 to 0.2)	0.0 (0.0 to 0.0)	−0.2 (−0.2 to −0.1)^b^	−0.1 (−0.2 to −0.1)	<.001
Nuts and seeds	0.2 (0.1 to 0.3)	0.3 (0.2 to 0.4)	0.1 (0.0 to 0.2)^d^	0.2 (0.1 to 0.3)	0.1 (0.0 to 0.1)	−0.1 (−0.2 to −0.0)^c^	−0.2 (−0.4 to −0.1)	<.001
Meat alternatives	0.1 (0.0 to 0.2)	0.2 (0.0 to 0.4)	0.1 (−0.1 to 0.3)	0.1 (0.0 to 0.3)	0.7 (0.4 to 0.9)	0.5 (0.3 to 0.7)^b^	0.5 (0.2 to 0.7)	.001
Dairy	0.6 (0.4 to 0.8)	0.4 (0.3 to 0.5)	−0.2 (−0.4 to 0.0)	0.8 (0.6 to 0.9)	0.1 (0.0 to 0.1)	−0.7 (−0.9 to −0.5)^b^	−0.5 (−0.8 to −0.2)	<.001
Full fat	0.2 (0.1 to 0.4)	0.1 (0.1 to 0.2)	−0.1 (−0.2 to 0.0)	0.4 (0.2 to 0.5)	0.1 (0.0 to 0.1)	−0.3 (−0.4 to −0.1)^b^	−0.2 (−0.4 to 0.0)	.07
Reduced fat	0.2 (0.1 to 0.3)	0.2 (0.1 to 0.3)	−0.0 (−0.1 to 0.1)	0.2 (0.1 to 0.3)	0.0 (0.0 to 0.0)	−0.2 (−0.3 to −0.1)^b^	−0.2 (−0.3 to −0.0)	.03
Nonfat	0.1 (0.1 to 0.2)	0.1 (0.0 to 0.1)	−0.1 (−0.2 to 0.0)	0.2 (0.1 to 0.3)	0.0 (0.0 to 0.0)	−0.2 (−0.3 to −0.1)^b^	−0.1 (−0.2 to −0.0)	.03
Dairy alternatives	0.2 (0.1 to 0.3)	0.1 (0.1 to 0.2)	−0.1 (−0.1 to 0.0)	0.2 (0.1 to 0.3)	0.2 (0.2 to 0.3)	0.1 (−0.0 to 0.2)	0.1 (−0.0 to 0.2)	.07
Added fat	0.3 (0.3 to 0.4)	0.6 (0.5 to 0.7)	0.3 (0.2 to 0.4)^b^	0.3 (0.3 to 0.4)	0.1 (0.1 to 0.1)	−0.2 (−0.3 to −0.1)^b^	−0.5 (−0.6 to −0.3)	<.001
Animal fat	0.0 (0.0 to 0.1)	0.0 (0.0 to 0.0)	−0.0 (−0.1 to −0.0)^d^	0.0 (0.0 to 0.1)	0.0 (0.0 to 0.0)	−0.0 (−0.0 to −0.0)^b^	0.0 (−0.0 to 0.0)	.98
Vegetable oil	0.3 (0.2 to 0.3)	0.6 (0.5 to 0.7)	0.3 (0.2 to 0.4)^b^	0.3 (0.2 to 0.3)	0.1 (0.1 to 0.1)	−0.2 (−0.2 to −0.1)^b^	−0.5 (−0.6 to −0.3)	<.001
Added sugar	0.2 (0.2 to 0.3)	0.2 (0.1 to 0.3)	−0.1 (−0.2 to 0.0)	0.2 (0.1 to 0.3)	0.2 (0.1 to 0.3)	−0.0 (−0.1 to 0.1)	0.1 (−0.0 to 0.2)	.23
Sugar-sweetened beverages	0.2 (0.1 to 0.2)	0.1 (0.0 to 0.1)	−0.1 (−0.2 to −0.0)^c^	0.2 (0.1 to 0.3)	0.0 (0.0 to 0.1)	−0.1 (−0.2 to −0.0)^c^	−0.1 (−0.2 to 0.1)	.38

^a^ *P* values for treatment effect size are from a 2-sample *t* test comparing mean changes between participants in each treatment arm.

^b^ *P* < .001 for within-group changes from baseline assessed by paired comparison *t* tests.

^c^ *P* < .01 for within-group changes from baseline assessed by paired comparison *t* tests.

^d^ *P* < .05 for within-group changes from baseline assessed by paired comparison *t* tests.

## Discussion

This secondary analysis of a randomized crossover trial found that total food costs were 25% lower on a vegan diet compared with a Mediterranean diet. The 19% reduction in food costs on a vegan diet from baseline is in line with a previous study showing a 16% reduction.^5^

Strengths of the present study include a randomized, crossover design, enabling a head-to-head comparison of food costs associated with both diets. The study also has limitations. Food consumption estimates were based on self-reported diet records in the US. Food cost estimates in the Thrifty Food Plan are conservative and exclude alcohol. The participants were volunteers and may not represent the general population. In conclusion, these findings suggest that total food costs decrease significantly in adopting a vegan diet compared both with baseline and with a Mediterranean diet.

## Notes

Supplement 1.
Trial Protocol and Statistical Analysis Plan

Supplement 2. **eFigure.** Enrollment of Participants and Completion of the Study

Supplement 3.
Data Sharing Statement
