# Supplementary material for: Food Costs of a Low-Fat Vegan Diet vs a Mediterranean Diet: A Secondary Analysis of a Randomized Clinical Trial
Source: JAMA Netw Open. 2024 Nov 18;7(11):e2445784. doi: 10.1001/jamanetworkopen.2024.45784 (PMC11574688; doi:10.1001/jamanetworkopen.2024.45784)
Supplement: Supplement 1. — Trial Protocol and Statistical Analysis Plan [file jamanetwopen-e2445784-s001.pdf]

Research Plan: A Randomized, Cross-Over Trial on the Effects of a Low-Fat Vegan Diet versus a Mediterranean Diet on Body Weight

Sponsor: Physicians Committee for Responsible Medicine

Protocol Number: Pro00029777

Version: 1.1

Summary

This randomized, cross-over trial aims to assess changes in body weight, plasma lipids, insulin sensitivity, and postprandial metabolism with a low-fat, plant-based diet and a Mediterranean diet, both followed for 4 months.

1. SPECIFIC AIMS AND OVERVIEW

1.1. Specific Aims

Specific Aim 1. This study tests the hypothesis that a low-fat vegan diet will result in greater weight changes than the Mediterranean diet.

Specific Aim 2. This study tests the hypothesis that a low-fat vegan diet will lead to greater reductions in total and LDL cholesterol concentrations than the Mediterranean diet.

Specific Aim 3. This study tests the hypothesis that a low-fat vegan diet will result in greater improvements in insulin sensitivity than the Mediterranean diet.

Specific Aim 4. This study tests the hypothesis that a low-fat vegan diet will lead to a greater increase in postprandial metabolism than the Mediterranean diet.

In addition. this study tests the explores the effects of both diets on cardiovascular function, as measured by concentrations of advanced glycation end-products and endothelial function, and on gut microbiome composition. Our hypothesis is that a low-fat vegan diet will lead to greater improvements than the Mediterranean diet. We hypothesize that weight loss will be independent on the participants' blood type.

1.2. Protocol Overview

In a 36-week cross-over trial, overweight adults will consume a low-fat vegan diet and a Mediterranean diet in two separate 16-week phases. Changes in body weight will be the primary dependent variable. In addition, plasma lipid concentrations, insulin sensitivity, and postprandial metabolism will also be assessed and changes over time will be compared between the two diets.

Participants will be randomly assigned to start either a low-fat vegan diet or a Mediterranean diet for 16 weeks. Both groups will receive weekly classes and support, and will be asked to make no changes to their exercise patterns for the study period. After a 4-week washout period, participants will cross over to the second dietary intervention for another 16 weeks.

1.3. Investigative Team

The project will be conducted by investigators from the Physicians Committee for Responsible Medicine (PCRM), a nonprofit 501(c)(3) organization located at 5100 Wisconsin Avenue, NW, Washington DC 20016, which conducts nutrition-related research. Its medical, nutrition, and research staff will oversee participant recruitment, screening, group assignment, nutrition teaching and monitoring, and data collection and analysis. Recruitment interviews, the dietary intervention, and all assessments will take place at its offices.

Dr. Robynne Chutkan, MD, will serve as a study consultant, as described below.

2. BACKGROUND AND SIGNIFICANCE

Excess body weight is a major contributor to many health problems, including diabetes, cardiovascular disease, orthopedic problems, and certain forms of cancer. In epidemiologic studies, individuals following vegan diets tend to have significantly lower body weights, compared with individuals following other dietary patterns.[1] In studies of overweight individuals, the adoption of a low-fat plant-based diet predictably reduces body weight, even in the absence of any specified limitation on energy intake.[2]

The mechanisms by which plant-based diets reduce body weight are not entirely clear. Previous studies have identified two possible explanations.[3] First, to the extent that vegan diets are low in fat and high in fiber, they have a relatively low energy density, which reduces energy intake. Second, a low-fat vegan diet may increase postprandial metabolism (the thermic effect of food). These observations suggest that the diet leads to weight loss by (1) reducing energy intake and (2) increasing postprandial energy output.

A prior study[3] including 64 overweight postmenopausal women randomly assigned to a low-fat vegan diet or a comparison diet based on the guidelines of the National Cholesterol Education Program for 14 weeks found that the vegan diet led to significantly greater weight loss (5.8 kg for the vegan group, compared with 3.8 kg for the comparison group). The vegan diet group also had a 16% increase in postprandial metabolism and an increase in insulin sensitivity that was significant within group, although not between groups. However, because the comparison group used an active diet and there was no untreated control group, that study was not able to show the degree to which a plant-based diet influences energy expenditure, compared with untreated participants.

Mediterranean-style diets improve plasma lipid concentrations. One study found that a Mediterranean-style diet experienced an 85% greater reduction in their LDL cholesterol levels compared to a control group.[4] Likewise, participants of the PREDIMED study following the Mediterranean diets also improved their LDL levels more than the control group.[5] However, another study failed to reduce LDL levels with a Mediterranean dietary intervention.[6]

In a systematic review, a Mediterranean diet led to greater long-term weight loss than low-fat diets and similar weight loss as comparator diets (such as low-carbohydrate diets, energy-restricted diets, etc) (range of mean changes: -3.8 to -10.1 kg).[7] The Mediterranean diet also led to greater improvements in fasting glucose and HbA1c levels in patients with type 2 diabetes than comparator diets. Participants following a Mediterranean diet experienced greater improvements in triglycerides but similar changes in LDL and HDL levels as those following the comparator diets. Thus, a Mediterranean diet can aid weight loss and blood lipids in overweight or obese individuals, to the same degree as other diets.

In research studies, the acceptability of plant-based diets appears to be similar to that of other therapeutic diets over both the short and long term, as indicated by rates of retention, diet adherence, and diet acceptance questionnaires.[8-11] Likewise, self-reports and objective measures indicate generally good adherence to the Mediterranean diet, suggesting its potential as a weight loss treatment. [12]

The present study provides a controlled comparison of a low-fat vegan diet and a Mediterranean-style diet by (1) providing regular support that is of identical intensity in the two study arms and (2) using participants as their own controls in a cross-over design.

3. RESEARCH DESIGN, RECRUITMENT, AND ASSESSMENTS

3.1. Overview of Research Design

In a randomized, cross-over trial, we will test the effects of a low-fat plant-based diet on body weight, blood lipids, insulin sensitivity, and postprandial metabolism in overweight adults over a 36-week period, using an analogous Mediterranean dietary intervention for comparison.

3.2. Key Personnel

Key personnel include:

Neal D. Barnard, MD, FACC, Principal Investigator, is an Adjunct Associate Professor of Medicine at the George Washington University and President of PCRM. He has been the Principal Investigator of several clinical trials, as noted herein.

Hana Kahleova, MD, PhD, is an endocrinologist and Director of Clinical Research at PCRM. She has been involved in several clinical trials in diabetes and insulin resistance.

Melissa Busta, RN, Jihad Alwarith, Nora Burgess, Katie Fletcher, Rosendo Flores, coordinate clinical research studies at PCRM.

Susan Levin, MS, RD, Lee Crosby, RD and Maggie Neola, RD, are Registered Dietitians at PCRM who provide nutrition instruction and participate in clinical assessments.

Richard Holubkov, PhD, is a biostatistician with the University of Utah, who works with PCRM on contract.

Robynne Chutkan, MD, is a gastroenterologist at Digestive Center for Wellness, who works with PCRM on contract.

3.3. Recruitment and Screening Procedures

Volunteers will be recruited through notices placed in waiting rooms of physicians' offices, letters sent to patients of medical practitioners, and advertisements placed in newspapers, on radio, and in buses in the Washington, DC, area, as well as social media postings. (**Appendix 1**)

Volunteers' initial calls will be directed to PCRM, where research staff will screen participants using a telephone screening script **(Appendix 2).** Research staff will explain the study, review participation criteria, and inquire about other motivations for volunteering, filling out a paper interview screening form for each person who calls. Volunteers who satisfy the participation criteria will be scheduled for group and/or individual information sessions. The names/identifiable information of volunteers who do not satisfy participation criteria will be destroyed (shredded) immediately. For these individuals, the research team will retain only de-identified demographic information and the reason for exclusion, for purposes of evaluating participation statistics.

At the group and/or individual information sessions (some volunteers may attend individually; others may be seen in groups), the investigators and research staff will explain the study and its scientific basis in detail and review participation criteria in simple, nontechnical terms. They will also provide instruction on filling out a diet record. Additional content will be determined by questions raised by volunteers, and may relate to study logistics, the recruitment process, the content of the vegan and Mediterranean diets and ease of following them, clinical assessments, or the weekly classes. To protect patient privacy, volunteer names will not be used at these meetings. Volunteers will have a chance to ask questions about the study and the informed consent process in private, and each volunteer will meet in private with study personnel at the conclusion of the group session, even if he or she has no questions. Volunteers who choose to complete the informed consent document **(Appendix 3)** will also be asked to complete a contact information form **(Appendix 4)** and a general medical history form **(Appendix**

5). Volunteers will be assigned identification numbers in the order in which they complete the informed consent document. These numbers will be used in place of identifying information for purposes of data collection, assessment, and analysis. During the screening process, prospective volunteers will be provided the study coordinator's phone number and contact information in order to be able to reschedule and cancel their appointments, if necessary.
Prospective volunteers will then be asked to complete a practice 3-day dietary record to demonstrate their ability to track nutrient intake for research purposes. A packet (**Appendix 6**) will be given to the participants instructing them on how to complete their 3-day diet record (**Appendix 6.1**). When completed, these records will be reviewed for completeness by a staff member certified in Nutrition Data System for Research.

Volunteers who have completed the informed consent process and practice dietary records and meet the study participation criteria will be asked to schedule individual appointments for baseline assessments. There, they will be asked to fill out new 3-day dietary records, including 3 consecutive days, including 2 weekdays and 1 weekend day. Volunteers can either submit questionnaires online, print, scan and email them, or send them through regular mail. Those who satisfactorily complete the baseline assessments and 3-day dietary records will be enrolled in the study.

The cost of all tests and procedures will be covered by PCRM. If any examination or test reveals that a participant has a medical condition that requires additional diagnostic tests or treatment, research staff will advise the participant of that fact, but will not provide such additional diagnostic tests or treatment. There is no cost for the weekly group sessions. Parking will be free during participants' visits to the PCRM office for assessments, meetings, and group sessions.

3.4. Inclusion and Exclusion Criteria

Inclusion criteria are as follows:

1. Men and women age ≥18 years of age
2. Body mass index 28-40 kg/m^2

Exclusion criteria are as follows:

1. Diabetes mellitus type 1, history of any endocrine condition that would affect body weight, such as thyroid disease, pituitary abnormality, or Cushing's syndrome
2. Smoking during the past six months
3. Alcohol consumption of more than 2 drinks per day or the equivalent, episodic increased drinking (e.g., more than 2 drinks per day on weekends), or a history of alcohol abuse or dependency followed by any current use
4. Use of recreational drugs in the past 6 months

5. Use within the preceding six months of medications that affect appetite or body weight, such as estrogens or other hormones, thyroid medications (unstable dose within the preceding 6 months), systemic steroids, antidepressants (tricyclics, MAOIs, SSRIs), antipsychotics, lithium, anticonvulsants, appetite suppressants or other weight-loss drugs, herbs for weight loss or mood, St. John's wort, ephedra, beta blockers
6. Pregnancy or intention to become pregnant during the study period, as verified by self-report
7. Unstable medical or psychiatric illness
8. Evidence of an eating disorder
9. Likely to be disruptive in group sessions
10. Already following a low-fat vegan diet or Mediterranean diet
11. Lack of English fluency
12. Inability to maintain current medication regimen
13. Inability or unwillingness to participate in all components of the study
14. Intention to follow another weight-loss method during the trial

3.5. Group Assignment

Participants will be told that, if accepted, they will be initially randomized to either a low-fat vegan diet or Mediterranean-style diet for 16 weeks. After a 4-week washout period, participants will cross over to the alternate dietary intervention for another 16 weeks. Accepted volunteers will be assigned to these groups using a computer-generated random-number table. Because assignment will be done simultaneously within each replication, allocation concealment is unnecessary.

3.6. Clinical Assessments

The following determinations will be made at baseline, week 16, week 20, and week 36, except as noted:

Assessments of Dietary Intake and Physical Activity

3-day dietary record. A 3-day dietary record, as described above, will be used to assess macro- and micronutrient intakes. Records will be analyzed using Nutrition Data System for Research software version 2016, developed by the Nutrition Coordinating Center (NCC), University of Minnesota, Minneapolis, MN, US, by a staff member certified by the NCC. Random 24-hour dietary recalls will be conducted by registered dietitians to determine compliance but will not be part of the final nutrient analysis.

The **International Physical Activity Questionnaire** short form assesses recent physical activity patterns. The method is highly reliable; an assessment of test-retest repeatability produced a correlation of 0.8.[13] **(Appendix 7)**

Assessments of Physical Health, Weight, and Metabolism

General status, symptoms, and medication accounting. Participants will be asked to report changes in their health and medication use.

Height. Height will be measured at baseline (only) with participants standing barefoot with their backs to a wall-mounted stadiometer and heels against the wall, recorded to the nearest 0.5 cm.

Body weight. With participants wearing light, indoor clothing but without shoes, body weight will be measured to the nearest 0.1 kg, using a digital scale. Body weight will also be assessed at each weekly group session, but only data from baseline, week 16, 20, and 36 will be included in the analysis.

Comprehensive Metabolic Panel. These values will be evaluated at baseline only.

Plasma cholesterol and triacylglycerol concentrations and **hemoglobin A1c** will be measured using standard methods.

Glucose Tolerance and Insulin Sensitivity. A standard meal test will be performed for three hours after an overnight fast. (Matsuda 1999)

Resting Energy Expenditure (REE). Participants will be asked to report to the laboratory within 60 minutes of waking and after a 12-hour fast. REE will be measured for 20 minutes through indirect calorimetry (Cosmed Quark RMR, Chicago, IL) utilizing a ventilated hood system. The laboratory temperature will be maintained at 23 degrees C throughout, and precautions will be taken to minimize any disturbances that could affect the metabolic rate.

Postprandial metabolism (thermic effect of food, TEF). After the REE determination, participants will be given a 720-kilocalorie test meal (Boost, Nestlé, Vevey, Switzerland) to be ingested within 10 minutes. Metabolic rate will be measured in the same manner as above for 20 minutes at 0, 30, 60, 120 and 180 minutes post-ingestion.

Body Composition. Body composition will be measured by dual energy x-ray absorptometry (Lunar iDXA, GE Healthcare; Madison, WI) with Encore® 2005 v.9.15.010 software. The iDXA can measure body composition with low x-ray exposure and short scanning time. The iDXA unit will be calibrated daily using the GE Lunar calibration phantom, and a trained operator will perform all scans following standard protocol for participant positioning. The iDXA is equipped with the CoreScan module (GE Healthcare, Madison, WI), which can also provide an estimate of visceral adipose tissue volume and mass.

Microbiome. The effect of dietary changes on the microbiome will be assessed through uBiome SmartGut microbiome screening test, by using advanced DNA sequencing to determine specific bacteria and other microorganisms. Study participants will be given their baseline, week 16, week 20 and, week 36 assessments. Study participants will

collect their stool sample and perform the uBiome test at home. They will then bring their packaged sample to the clinic to be shipped in one shipment to uBiome for analysis. Results will be sent to Digestive Center for Wellness, where Dr. Robynne Chutkan will interpret the results. With this, we are able to track any changes in microbiome related to dietary changes.

Endothelial function. Endothelial function will be assessed with the use of the itamar EndoPAT. EndoPAT quantifies the endothelium-mediated changes in vascular tone, elicited by a 5-minute occlusion of the brachial artery (using a standard blood pressure cuff). When the cuff is released, the surge of blood flow causes an endothelium-dependent flow mediated dilatation. The dilatation is captured by EndoPAT as an increase in the PAT signal amplitude. The PAT signal used in the EndoPAT is measured from the fingertip by recording finger arterial pulsatile volume changes. Results of the 15-minute test are automatically calculated and an EndoScore is generated, which indicates the present state of endothelial health. We will assess the endothelial function at baseline, week 16, week 20, and week 36.

Advanced Glycation Endproducts. Advanced Glycation End-products will be measured using the Advanced Glycation End-products (AGE) Reader mu by Diagnoptics. AGE's are produced through the glycation of glucose and proteins, resulting in artery stiffness. Elevated blood sugar from diabetes can result in an accumulation of AGE's, accelerating the development of cardiovascular complications. AGE Reader produces ultra-violet light to excite AGE's in human skin. AGE Reader measures the skin fluorescence to quantify the tissue accumulation of AGE's. This measurement is used to determine the corresponding level of AGE's in the blood vessel wall. A cardiovascular risk report is generated after the 12 second assessment and displayed as a color-coded graph (ranging from No Cardiovascular Risk to Definite Cardiovascular Risk). AGE levels will be measured at baseline, week 16, week 20, and week 36.

Blood Type*:* The Physicians Committee will contact all participants to see if they know of their blood type and are willing to disclose the information.

Table 1: Study Procedures Schedule

Week	0	16	20	36
3-day diet record	√	√	√	√
International Physical Activity Questionnaire	√	√	√	√
Clinical status and symptoms	√	√	√	√
Medication use	√	√	√	√
Height	√			
Body weight*	√	√	√	√
Comprehensive Metabolic Panel (CMP)	√			
Plasma lipids and lipoproteins	√	√	√	√
A1c	√	√	√	√

High Sensitivity C-Reactive Protein	√	√	√	√
Glucose tolerance testing	√	√	√	√
REE	√	√	√	√
Postprandial metabolism (TEF)	√	√	√	√
Body composition	√	√	√	√
Microbiome analysis (uBiome)	√	√	√	√
Endothelial function (EndoPAT)	√	√	√	√
Advanced Glycation Endproducts (AGE)	√	√	√	√

4. INTERVENTION PROCEDURES

4.1. Intervention Diets

The interventions for the low-fat vegan diet and Mediterranean-style diet are described below. Both are to be followed ad libitum, that is, there will be no restriction on energy intake in either group and there will be no attempt to create isocaloric interventions.

The Low-Fat Vegan Diet Group. According to the Academy of Nutrition and Dietetics, vegan and vegetarian diets meet all nutritional requirements when appropriately planned.[14] The diet consists of whole grains, vegetables, legumes, and fruits, with no restriction on energy intake. Animal products and added oils will be excluded. In choosing grain products and starchy vegetables (e.g., bread, potatoes), participants will be encouraged to select those retaining their natural fiber and having a glycemic index <70, using tables standardized to a value of 100 for glucose. No meals will be provided. Participants will handle their own food preparation and purchases, with guidance from the research team, with no restriction on energy intake.

The diet derives approximately 10% of energy from fat, approximately 10-15% of energy from protein, and the remainder from complex carbohydrates. The diet will provide approximately 30-40 grams of fiber per day. It is generally adequate in all nutrients except vitamin B_{12}.

Participants will be provided with a commercially available supplement containing 100 micrograms of vitamin B_{12} and asked to take it daily during the study. Should they wish to continue the diet thereafter, they will be counseled to use any standard multivitamin or other reliable source of vitamin B_{12}.

An advantage of studies such as this one, which include volunteers who are not confined to a metabolic ward or otherwise restricted, is that they can readily translate to nonclinical settings. A disadvantage is that they include a degree of uncertainty as to the extent to which participants have adhered to their prescribed diets. While this uncertainty cannot be entirely eliminated, several measures will be taken to maximize

dietary adherence, based on the current investigators' published review of factors associated with dietary compliance in clinical trials. Stricter limits on fat intake, frequent monitoring of reported dietary intake, family involvement, group support, and the use of vegetarian diets are associated with a greater degree of dietary change.[15]

The Mediterranean Diet Group. Participants will be asked to follow the Mediterranean-style diet as developed for PREDIMED study. [16] The diet consists of ≥2 daily serving of vegetables, ≥2-3 daily servings of fresh fruits, ≥3 weekly servings of legumes, ≥3 weekly servings of fish or seafood, ≥ 3 weekly serving of nuts or seeds, select white meats (with visible fat removed) instead of red meats, with no restriction on energy intake. Participants will be asked to eliminate or limit the consumption of cream, butter, margarine, processed meats, sweetened beverages, pastries, and processed snacks. Nuts, eggs, seafood, low-fat cheese, chocolate (≥ 50% cocoa) and whole-grain cereals may be consumed ad libitum, while cured ham, red meat, and fatty cheeses will be limited to ≤1 serving per week. Participants will be asked to include 50g of extra virgin olive oil (approximately 4 tbsp. or ¼ cup) in their daily routines. Participants will handle their own food preparation and purchases, with guidance from the research team. Extra virgin olive oil will be provided, but no meals will be provided.

Both groups: For both groups, alcoholic beverages will be limited to one per day for women, and two for men.

4.2. Dietary Instruction and Group Meetings

Participants will be asked to attend weekly, one-hour group sessions for support and education. **(Class Curriculums, Appendix 8, Appendix 9).** No weekly support or education will be provided to the participants during the 4 week washout period, between weeks 16 and 20.

All group sessions will be conducted by a registered dietitian, nurse, physician, cooking instructor, or research staff and will include information on nutrition, meal planning, shopping, food preparation techniques, recipes, and everyday dietary challenges, such as dining out and healthful snacking. The classes will also include education on topics such as maintaining a healthy weight, cholesterol, hypertension, diabetes, and other health issues.

For some sessions, participants will be encouraged to bring a spouse, partner, family member, or friend. To facilitate interaction between diet instructors and participants, classes will be conducted in sections of approximately 15 participants.

The curriculum is based on the investigators' prior studies and uses concepts from the Health Belief Model developed by researchers with the Public Health Service and adapted by others.[17] This model describes constructs that predict health-related behaviors and should be considered when planning behavioral change strategies. These include perceived susceptibility, severity, benefits, and barriers, as well as cues to action, and self-efficacy. Our participants are already aware that they are overweight

and may benefit from diet changes. Nonetheless, they need help in overcoming barriers and gaining confidence in their ability to implement new dietary habits. We have therefore focused the content of the weekly support group sessions on integrating practical skills (e.g., menu planning, food preparation, dining out, healthful snacking) with their growing understanding of how dietary choices affect health. In order to facilitate individual experience with the prescribed diet, practical skills are presented early, while intellectual understanding of more complex health issues (e.g., how diet affects heart disease risk) is presented later. Each group session includes time for participants to discuss their successes and challenges, and group problem-solving is encouraged.

The study does not seek to separate the effects of the diet from those of regular group support. Rather, group support is a means of facilitating adherence. It should also be emphasized that the goal of this study is not to construct isocaloric dietary interventions.

4.3. Exercise and Medication Use

Participants in both groups will be asked to keep their level of physical exercise and use of medications constant and to add no new nutritional supplements to their current medication regimens, except as recommended by their personal physicians.

4.4. Intervention Fidelity and Dietary Adherence

Individual meetings. During the initial individual meal-planning meetings with the study participants, dietitians will follow a set agenda which will cover the guidelines of the given diet.

Group meetings. To maintain intervention fidelity, the group leaders will follow a set course curriculum, using an agenda for each session and keeping a checklist of major content items to be covered at each meeting.

Dietary Adherence. Each participant will complete diet records at regular intervals using the methods described above. In addition, 24-hour multi-pass dietary recalls will be used to assess dietary adherence to assist study personnel in working with individuals who need additional teaching or support. The 24-hour recalls will be performed either by telephone or in person at week 3, week 8, week 23, and week 28. These recalls will not be subjected to statistical analysis, but will allow the investigators to check for poor adherence. Such recalls have the advantage that they can be conducted at unscheduled times and over the telephone, and so are not subject to the planning and preparation required for food records.[18] In cases where participants appear to be deviating from the prescribed diet, additional dietary counseling will be provided.

4.5. Participant Retention

Participants’ interests for volunteering will be ascertained during screening. Those with reasons for volunteering other than a desire to improve their health or to advance scientific understanding may be rejected. The exclusion criteria also eliminate individuals with a history of unresolved substance abuse, which may influence retention.

Participants will be instructed that attendance at meetings is essential to study participation. The research team will take attendance at each meeting. The research staff will make phone calls to participants who do not attend.

In the weekly meetings, group support will be facilitated through group discussions and encouragement to share successes and difficulties with the prescribed diet. Meeting content will remain varied, including nutrition lectures, health education, cooking demonstrations, and opportunities to taste food. Family members will be invited to certain support group sessions. A voluntary listserv will allow participants to exchange information, recipes and ideas between meetings. Only participants, study coordinators, and the PI will be allowed to post on the listserv and the content of the listserv will be accessible only by them.

Participants who complete all assessments at weeks 0, 16, 20 and 36 will be paid $100 at completion of both their week 16 and week 36 assessments.

4.6. Biological Specimen Handling Procedures

Samples for the study endpoints will be drawn by a registered nurse, employed by PCRM, and will be processed at Sunrise Medical Laboratories using standard procedures.

5. STATISTICAL PROCEDURES

5.1. Power Analysis

Power Analysis for Overall Study

Sample size will be based on the change in body weight, previously observed with a plant-based diet, compared with a Mediterranean diet.[3] The current power analysis is based on an alpha level of 0.05.

Based on previous studies, the expected changes in body weight are -0.3 (95% CI -0.61 to 0.09) kg on the Mediterranean diet and -6.5 (95% CI -8.9 to -4.1) kg on the low-fat vegan diet in 16 weeks. For 90% power to detect a significant treatment effect, we need 24 participants to complete both interventions in a cross-over fashion. Assuming an attrition of 20%, the required sample size is 30 total for 90% power.

5.2. Data Management

All laboratory samples, reports, questionnaires, and data sheets will be coded with participant identification numbers, rather than names. Laboratory reports will be delivered to the PCRM office at 5100 Wisconsin Avenue, Washington, D.C., where they, along with all other history and data forms, will be maintained in individual participant files in a locked cabinet.

Data will be promptly entered into the data tables at PCRM using Microsoft Excel. Two research staff members will check the tables for accuracy against the original documents. Data tables will be routinely copied onto back-up files and stored for safety on an off-site, passcode-protected, secure server. Data grids will be sent electronically to the biostatistician for analysis. Registered dietitians will be provided with information on usual ranges for nutrients or intakes of interest and asked to check their original data and analysis for errors if they fall outside of these ranges.

5.3. Statistical Analysis

Descriptive statistics for all demographic variables and clinical measures will be calculated for each group. To determine if there are statistically significant differences between the 2 groups at baseline, t-tests will be calculated for continuous measures and chi squares will be calculated for categorical measures. Regardless of any differences, baseline values for key outcome variables will be included as covariates in the main assessments of the effect of diet in the multivariate analysis of covariance. An alpha of 0.05 will be used for all statistical tests.

For nutrient intake and physical measures, descriptive statistics (means, standard deviations, tests for normality) will be calculated. If data are normally distributed, parametric tests for significant effects will be used; for non-normally distributed variables, non-parametric tests will be used.

The initial test of the hypotheses will be examined by performing t-tests for independent samples on the difference score denoting the change from baseline to the reporting period. For missing data in a reporting period, values from the previous period will be brought forward. For body weight, drop-outs will be considered to have returned to baseline weights.

5.4. Assessment of Diet Adherence. Participants will be described as adherent or non-adherent to vegan diet based on whether they met the following criteria: absence of proscribed foods reported on 24-hour recalls and diet records, saturated fat <5% and total fat <25% of energy, and average daily cholesterol intake <50 mg on 3-day dietary records. Participants will also complete a "Vegan Adherence Questionnaire" (**Appendix 10**). Adherence to the Mediterranean diet will be determined using the "Quantitative 14-item Score of Compliance with the Mediterranean Diet.", with a score of 0 points

indicating minimum adherence and 14 points indicating maximum adherence (**Appendix 11**).

For drop-out rates, we will determine if there are between-group differences, using chi-square.

5.5. Assessment of Medication use. Any changes to lipid-lowering medications will be classified as a net increase, net decrease, or mixed (changes in opposing directions for 2 or more medications). Using chi-square, we will determine whether there are differences in medication changes between the 2 groups.

6. TIME LINE AND PARTICIPANT FLOW

6.1. Time Line

Recruitment will take place between September 2018 – January 2019. The intervention, including weekly meetings, will take place between January and October 2019 for a total of 36 weeks. Subsequent cohorts would follow the same timeline.

6.2. Participant Flow Based on Power Analysis

Baseline assessments will occur in January 2019 for the first dietary intervention.

The group sessions for the first dietary intervention will be held for 16 weeks, from January through May 2019.

The 16-week assessments will occur in May 2019 for the first dietary intervention.

Participants will undergo a 4-week washout period, following the first dietary intervention, from May through June.

Week 20 Assessments will occur following the 4-week washout in June 2019.

The group sessions for the second dietary intervention will be held for 16 weeks, from June through October 2019.

The 36-week assessments will occur in October 2019 for the second dietary intervention.

7. PROTECTION OF HUMAN RESEARCH PARTICIPANTS

7.1. Risks to the Subjects

Sources of Materials: Participants will be asked to complete questionnaires, provide blood samples, and have several physical assessments.

Human Subjects Involvement and Characteristics: The proposed research will include participants at least 18 years of age.

Potential Risks: Participation in the study entails the following risks:

1. Blood draws can cause transient pain, occasionally cause bruising, and may cause bleeding.

2. A well-planned vegan diet provides all the nutrients people need except for vitamin B12. People with a vitamin B12 deficiency may suffer from anemia and neurologic damage.

3. Loss of confidential information.

7.2. Adequacy of Protection against Risks

Recruitment and Informed Consent: Participants will be informed of the study's goals and procedures and review the inclusion and exclusion criteria. Volunteers who appear to meet the criteria for participation will be invited to a group or individual interview with the principal investigator and the study coordinator, who will explain the study in detail, answer questions, and provide a written consent form, as approved by the IRB. Participants will have the opportunity to ask any questions individually in a private setting and may take as much time as they would like to review the informed consent document. The consent form will be signed by the volunteer participant and study coordinator. The principal investigator will certify that the research study has been explained to the volunteer, including the purpose, procedures, possible risks, and potential benefits associated with participation and that any questions have been answered to the volunteer's satisfaction.

Participants' personal physicians will be notified of their involvement in the study and that the investigators will not manage any aspects of their medical care.

To maintain confidentiality, all laboratory specimens, questionnaires, forms, and data sheets will identify participants by their assigned numbers only. Data and safety monitoring are described below.

Phlebotomy risks. All blood draws will be carried out by an experienced registered nurse, employed by PCRM.

Vitamin B12 deficiency. All vegan diet group participants will be given a supply of vitamin B12, 100 micrograms, and will be asked to take it daily. Participants will also be counseled to continue B12 supplementation if they plan to continue following a vegan diet.

Loss of confidential information. We will make every effort to keep all research records private to the extent allowed by law. We will use an identification number on forms, instead of identifiable information. All study documents will be kept in locked filing cabinets and in password protected electronic files at PCRM's office. Information we learn from this study may be shared at scientific or medical meetings and may be published, but participants will not be personally identified.

Our protocol also includes the following safeguards:

1. All participants will remain under the care of their personal healthcare providers.

2. All participants will continue on the medications they were using at study entry, unless modified by their personal physician(s).

We therefore believe that the risks to participants in a dietary intervention trial are minimal, while the scientific and public health merit of such an investigation is high. By studying the benefits of a dietary intervention, we hope to obtain valuable research data.

Confidentiality. To maintain confidentiality, all laboratory specimens, questionnaires, forms, and data sheets will identify participants by their assigned numbers only. Data and safety monitoring are described below.

7.3. Potential Benefits of the Proposed Research to the Participants and Others

Given that, over the long run, excess body weight contributes to morbidity and mortality, the dietary instruction and consistent support provided may be of substantial benefits for the study participants.

7.4. Importance of the Knowledge to Be Gained

Weight problems are extremely common, and gaps remain in our understanding of how intervention diets work. This study is founded on clear theoretical constructs and compelling previous data on both the efficacy and acceptability of the experimental interventions, as well as preliminary findings on its mechanisms of action. It investigates what may be a major advance in the understanding of the role of diet in weight control. The risks to participants are small, and the potential benefits are significant.

7.5. Assessment and Reporting of Adverse Events

An adverse event is any adverse physical or clinical change experienced by a participant. This includes the onset of new symptoms and the exacerbation of pre-existing conditions. In order to avoid bias in eliciting reports of adverse events, participants will be asked, during assessments at the end of each study period, "Have you had any new symptoms, injuries, illness or side effects or worsening of pre-existing conditions?" at each visit.

All adverse events will be recorded in the participant's record and on the IRB continuing review form. The severity of the adverse event will be assessed, and actions/outcomes (e.g., hospitalization, discontinuation of therapy, etc.) will also be recorded.

Any actions taken and follow-up results will also be recorded on the appropriate page of the IRB continuing review form, as well as in the participant's record. Follow-up laboratory results will be filed with the participant’s record. All adverse events occurring at a site will be reported by the investigator to the IRB according to the Data and Safety Monitoring Plan, described below.

The following definitions will be used:

Minimally serious:	Awareness of sign, symptom, or event, but easily tolerated.
Somewhat Serious:	Discomfort enough to cause interference with usual activity and may warrant investigation.
Very Serious:	Incapacitating, with inability to do usual activities, or significantly affects clinical status, and warrants intervention.
Life-threatening:	Immediate risk of death.

The research team will also assess the relationship of any adverse event to the study intervention, based on available information, using the following guidelines:

0 = Unlikely	No temporal association, or the cause of the event has been identified, or the study interventions cannot be implicated.
1 = Possibly	Temporal association, but other etiologies are likely the cause; however, involvement of the study interventions cannot be excluded.
2 = Probably	Temporal association or other etiologies are possible, but unlikely.

7.6. Serious Adverse Events (SAEs)

All serious adverse events, whether or not deemed intervention-related or expected, will be reported by telephone to the Safety Officer within 24 hours (one working day) of the time they become known. A written report will follow as soon as possible, including a full description of the event and any sequelae. This includes serious events that occur any time after the inclusion of the patient in the study until completion of the last visit. A serious adverse event report will also be sent via fax to the IRB chair.

A serious adverse event is any event that falls in any of the following categories:

- Fatal
- Life-threatening (the patient was at immediate risk of death from the AE as it occurred)
- Significantly or permanently disabling
- Requires hospitalization or prolongs hospitalization

Important medical events that may not result in death, be life-threatening, or require hospitalization may be considered serious adverse events when, upon appropriate medical judgment, they may jeopardize the patient and may require medical or surgical intervention to prevent one of the outcomes listed in the definition. The death of any patient during the study, regardless of the cause, will be reported within 24 hours by telephone to the Safety Officer and IRB. A full written report will follow as soon as possible. If an autopsy is performed, a copy will be provided to the Safety Officer and IRB.

Reports of all serious adverse events, including deaths, will be communicated to the IRB in accordance with local laws and regulations.

7.7. Action plan if a subject becomes severely depressed or suicidal during the course of the study

Participants with a history of severe mental illness (with current unstable status), such as severe depression or suicidality, will not be enrolled in the study as indicated in the exclusion criteria. If a participant becomes severely depressed during the course of the study, he or she will be referred to see his or her primary care physician or psychiatrist and to seek medical care. The event will be recorded and reported to the PI and Safety Officer immediately. The PI will notify the IRB within 24 hours of recognition of the event by study personnel. All non-serious events will be reported and reviewed by the PI within one week. Study personnel will inform the primary care physician of all events occurring in his/her patients within 48 hours of recognition of the event.

If a participant becomes suicidal during the course of the study, he or she will be instructed to call 911 and seek emergency medical care. The incident will be recorded and reported to the PI and Safety Officer immediately. The PI will notify the IRB within 24 hours of recognition of the event by study personnel. All non-serious events will be reported and reviewed by the PI within one week. Study personnel will inform the primary care physician of all events occurring in his/her patients within 48 hours of recognition of the event.

7.8. Data and Safety Monitoring Plan

Data and Safety Monitoring functions will be performed by the principal investigator (PI, Neal D. Barnard, M.D.), study coordinators (Melissa Busta, RN, Jihad Alwarith, Nora Burgess, Katie Fletcher, Rosendo Flores), study statistician, and a Safety Officer who is a physician who is not part of the research staff and has no role in care of the

participants. The Safety Officer will have no scientific, financial, or other conflict of interest related to the trial. Prior to the study onset, the study statistician and Safety Officer will review the research protocol, informed consent documents, and plans for data and safety monitoring.

During the recruitment phase, the study coordinator and PI will review enrollment weekly. At monthly intervals, they will produce a report describing the study's progress, including accrual, demographics, thoroughness of baseline data, subject status (reporting concurrent illnesses, withdrawal of consent, or loss to follow-up), and adherence to participation criteria, informed consent procedures, and the study protocol. The reports will be submitted to the study statistician and Safety Officer.

Prior to participant randomization, a full history of each participant's pre-existing symptoms and medical problems will be recorded. During each monitoring visit, study participants will be asked if any medical symptom, problem, or event has occurred or if there has been any change in pre-existing symptoms.

All serious events (hospitalization, serious illness, or disability) will be recorded and reported to the PI and Safety Officer. The PI will notify the IRB within 24 hours of recognition of the event by study personnel. All non-serious events will be reported and reviewed by the PI within one week. Study personnel will inform the primary care physician of all events occurring in his/her patients within 48 hours of recognition of the event.

At monthly intervals, the study coordinator and PI will prepare and submit to the study statistician and Safety Officer a report covering each of the following areas: (1) performance (including adherence to the study protocol and maintenance of data integrity and confidentiality), (2) safety (including abnormal laboratory values, adverse events, serious adverse events, deaths, and disease- or treatment-specific events), and (3) treatment effects, including medication changes.

The study statistician and Safety Officer will review each safety report within one week of receipt. The study statistician will review these reports to assess whether event rates are of statistical concern and, if so, will alert the Safety Officer, the PI, and the IRB. The study statistician and Safety Officer will also consider factors external to the study, e.g., new scientific developments, that may affect the safety of participants or the conduct of the trial.

The Safety Officer will make recommendations as necessary to the PI. If the Safety Officer recommends a study change for patient safety or for ethical reasons, or if the study is closed early due to slow accrual, the PI will be responsible for implementing the recommendations as expeditiously as possible. If the PI does not concur with any recommendation of the Safety Officer, both will be responsible for reaching a mutually acceptable decision.

7.9. Stopping Rules

At the conclusion of the 36-week intervention period, the study statistician will prepare a report on clinical changes and adverse events for presentation to the PI and the Safety Officer. If evidence available at that point clearly shows either (1) an effect of the intervention diet on body weight, lipid profile, insulin sensitivity and postprandial metabolism or (2) harm associated with the intervention diet, the Safety Officer may recommend early termination of the study.

8. INCLUSION OF WOMEN, MINORITIES, AND CHILDREN

8.1. Inclusion of Women

The participation criteria, cited above, include both men and women. Recruitment procedures are expected to yield roughly equal numbers of men and women.

8.2. Inclusion of Minorities

The U.S. Census Bureau reports both race and ethnicity, the latter term used primarily to denote self-identification as Hispanic or non-Hispanic. According to the 2015 Census Bureau, races were represented in Washington, DC, as follows: 48.3% Black, 44.1% White, 4.2% Asian, 0.6% American Indian/Native American, 0.2% Native Hawaiian or other Pacific Islander; 2.7% 2 or more races. In addition, 10.6% of the population identified themselves as Hispanic.[19] In our prior studies, the respondent populations have been demographically diverse, reflecting the profile of the greater Washington, D.C area.

8.3. Inclusion of Children.

Persons less than 18 years of age will not be included in the study because they have insufficient control over the dietary choices that are essential to meaningful participation.

9. BRIEF STATEMENT OF ANTICIPATED OUTCOMES

This study aims to test hypotheses that are potentially important for individual and public health. It will improve our understanding of the treatment of weight problems and will also have practical implications for reducing the medical, personal, and economic costs associated with obesity. Anticipated outcomes for participants include beneficial changes in body weight, insulin sensitivity, and serum lipid concentrations.

LITERATURE CITED

[1] Tonstad, S, Butler T, Yan R, Fraser GE.Type of vegetarian diet, body weight and prevalence of type 2 diabetes. *Diabetes Care*. 2009;32:791-6.

[2] Barnard ND, Levin SM, Yokoyama Y. A systematic review and meta-analysis of changes in body weight in clinical trials of vegetarian diets. *J Acad Nutr Diet*. 2015 Jun;115(6):954-69.

[3] Barnard ND, Scialli AR, Turner-McGrievy G, Lanou AJ, Glass J. The effects of a low-fat, plant-based dietary intervention on body weight, metabolism, and insulin sensitivity. *Am J Med* 2005;118:991-997.

[4] Vincent-Baudry S, Defoort C, Gerber M, et al. The Medi-RIVAGE study: reduction of cardiovascular disease risk factors after a 3-mo intervention with a Mediterranean-type diet or a low-fat diet. *Am J Clin Nutr*. 2005;82(5):964-971.

[5] Estruch R, Ros E, Salas-Salvadó J, et al. Primary Prevention of Cardiovascular Disease with a Mediterranean Diet Supplemented with Extra-Virgin Olive Oil or Nuts. N *Engl J Med*. 2018;378(25):e34.

[6] de Lorgeril M, Salen P, Martin JL, Monjaud I, Delaye J, Mamelle N. Mediterranean diet, traditional risk factors, and the rate of cardiovascular complications after myocardial infarction: final report of the Lyon Diet Heart Study. Circulation. 1999;99(6):779-785.

[7] Mancini JG, Filion KB, Atallah R, Eisenberg MJ. Systematic Review of the Mediterranean Diet for Long-Term Weight Loss. *Am J Med*. 2016;129(4):407-415.e4.

[8] Barnard N, Scherwitz L, Ornish D. Adherence and acceptability of a lowfat vegetarian diet among patients with cardiac disease. *J Cardiopulmonary Rehabil* 1992;**12**:423-31.

[9] Barnard N, Scialli A, Bertron P, Hurlock D, Edmonds K. Acceptability of a therapeutic low-fat, vegan diet in premenopausal women. *J Nutr Educ* 2000;**32**:314-9.

[10] Barnard ND, Scialli AR, Turner-McGrievy G, Lanou AJ. Acceptability of a low-fat vegan diet compares favorably to a step II diet in a randomized, controlled trial. *Journal of cardiopulmonary rehabilitation* 2004;**24**(4):229-35.

[11] Barnard ND, Gloede L, Cohen J, et al. A low-fat vegan diet elicits greater macronutrient changes, but is comparable in adherence and acceptability, compared with a more conventional diabetes diet among individuals with type 2 diabetes. *J Am Diet Assoc* 2009;**109**(2):263-72.

[12] Estruch R, Ros E, Salas-Salvadó J, et al. Primary Prevention of Cardiovascular Disease with a Mediterranean Diet Supplemented with Extra-Virgin Olive Oil or Nuts. N *Engl J Med*. 2018;378(25):e34.

[13] Craig CL, Marshall AL, Sjostrom M, et al. International physical activity questionnaire: 12-country reliability and validity. *Medicine and science in sports and exercise* 2003;**35**(8):1381-95.

[14] Position of the American Dietetic Association and Dietitians of Canada: Vegetarian diets. *J Am Diet Assoc* 2003;**103**(6):748-65.

[15] Barnard N, Akhtar A, Nicholson A. Factors that facilitate dietary change. *Arch Fam Med* 1995;**4**:153-8.

[16] Estruch R, Ros E, Salas-Salvadó J, et al. Primary Prevention of Cardiovascular Disease with a Mediterranean Diet Supplemented with Extra-Virgin Olive Oil or Nuts. N *Engl J Med*. 2018;378(25):e34.

[17] Becker M. The health belief model and personal health behavior. *Health Education Monographs* 1974;**2**:324-473.

[18] Buzzard I, Faucett C, Jeffery R, et al. Monitoring dietary change in a low-fat diet intervention study: advantages of using 24-hour dietary recalls vs food records. *J Am Diet Assoc* 1996;**96**:574.

[19] U.S. Census Bureau. Quick Facts. District of Columbia. Internet: http://www.census.gov/quickfacts/table/RHI125215/11, accessed August 22, 2016.
