# Supplementary material for: Food Costs of a Low-Fat Vegan Diet vs a Mediterranean Diet: A Secondary Analysis of a Randomized Clinical Trial
Source: JAMA Netw Open. 2024 Nov 18;7(11):e2445784. doi: 10.1001/jamanetworkopen.2024.45784 (PMC11574688; doi:10.1001/jamanetworkopen.2024.45784)
Supplement: Supplement 2. — eFigure. Enrollment of Participants and Completion of the Study [file jamanetwopen-e2445784-s002.pdf]

Supplementary Online Content

Kahleova H, Sutton M, Maracine C, et al. Food costs of a low-fat vegan diet vs a Mediterranean diet: a secondary analysis of a randomized clinical trial. *JAMA Netw Open.* 2024;7(11):e2445784. doi:10.1001/jamanetworkopen.2024.45784

eFigure. Enrollment of Participants and Completion of the Study

This supplementary material has been provided by the authors to give readers additional information about their work.

© 2024 Kahleova H et al. *JAMA Network Open.*

eFigure. Enrollment of Participants and Completion of the Study

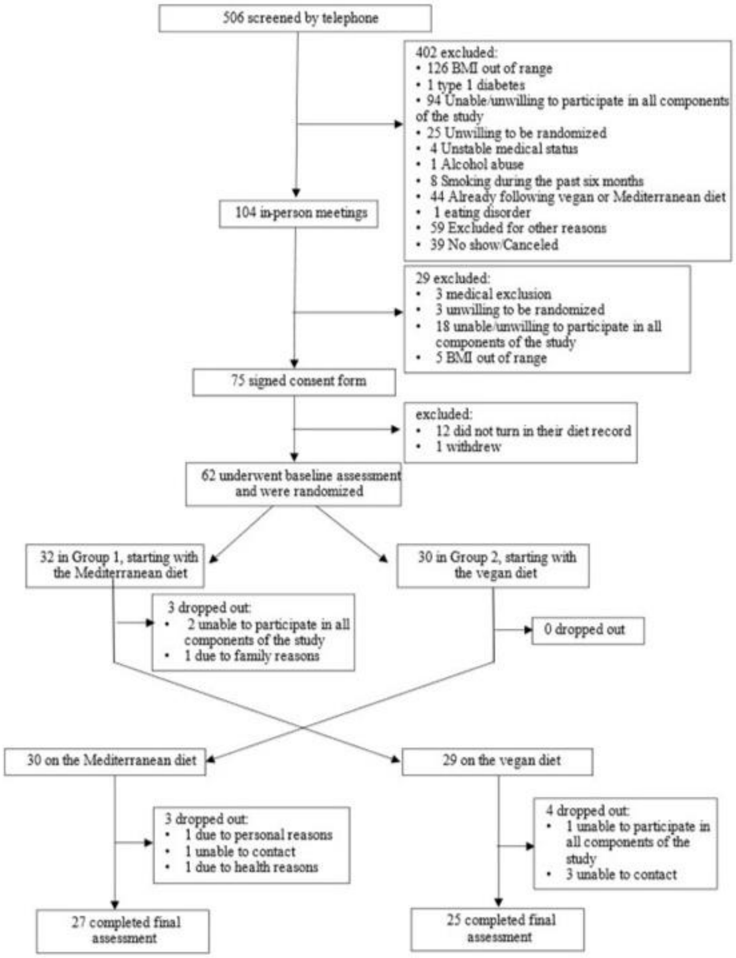

© 2024 Kahleova H et al. *JAMA Network Open.*
