# Supplementary material for: Food Costs of a Low-Fat Vegan Diet vs a Mediterranean Diet: A Secondary Analysis of a Randomized Clinical Trial
Source: JAMA Netw Open. 2024 Nov 18;7(11):e2445784. doi: 10.1001/jamanetworkopen.2024.45784 (PMC11574688; doi:10.1001/jamanetworkopen.2024.45784)
Supplement: Supplement 3. — Data Sharing Statement [file jamanetwopen-e2445784-s003.pdf]

Data Sharing Statement

Kahleova. Food Costs of a Low-Fat Vegan Diet vs a Mediterranean Diet. *JAMA Netw Open*. Published November 18, 2024. doi:10.1001/jamanetworkopen.2024.45784

Data
Additional Information: ClinicalTrials.gov number, NCT03698955
Data available: Yes
Data types: Deidentified participant data
How to access data: Data will be made available upon request at hana.kaheova@gmail.com.
When available: With publication

Supporting Documents
Document types: Statistical/analytic code, Informed consent form
How to access documents: The documents will be made available upon request at hana.kaheova@gmail.com.
When available: With publication

Additional Information
Who can access the data: Data will be made available to researchers whose proposed use of the data has been approved.
Types of analyses: Systematic reviews and meta-analyses.
Mechanisms of data availability: After approval of a proposal.
